# Parameter Optimisation in 3D Extrusion Printing of Polyhydroxybutyrate Using Design of Experiment Methodology †

**DOI:** 10.3390/jfb17020090

**Published:** 2026-02-12

**Authors:** Mingzu Du, Giuseppe Tronci, Xuebin B. Yang, David J. Wood

**Affiliations:** 1 Biomaterials and Tissue Engineering Research Group, School of Dentistry, University of Leeds, Leeds LS9 7TF, UK; dnmdu@leeds.ac.uk (M.D.);; 2 Clothworkers’ Centre for Textile Materials Innovation for Healthcare (CCTMIH), School of Design, University of Leeds, Leeds LS2 9JT, UK; 3 Bragg Centre for Materials Research, University of Leeds, Leeds LS2 9JT, UK

**Keywords:** extrusion printing, polyhydroxybutyrate, DoE, printability

## Abstract

This study systematically optimised extrusion-printing parameters for polyhydroxybutyrate (PHB) using a Design of Experiment (DoE) approach to improve printability and construct fidelity. A five-factor DoE was conducted to evaluate the individual and interactive effects of printhead temperature, printing pressure, printing speed, bed temperature, and cartridge heating time on the dimensional accuracy of printed constructs. The resulting regression model enabled the identification of statistically significant main and interaction effects among processing variables. An optimised parameter set (printhead temperature 145 °C, pressure 150 kPa, speed 15 mm s^−1^, bed temperature 25 °C, and cartridge heating time 120 s) enabled the fabrication of PHB scaffolds with substantially improved shape fidelity, which was experimentally validated using verification prints. These results demonstrate that a DoE-based optimisation strategy provides a robust and efficient route for rationally tuning PHB extrusion-printing conditions, thereby enhancing process reliability for scaffold fabrication in regenerative medicine applications.

## 1. Introduction

**Figure 1:**
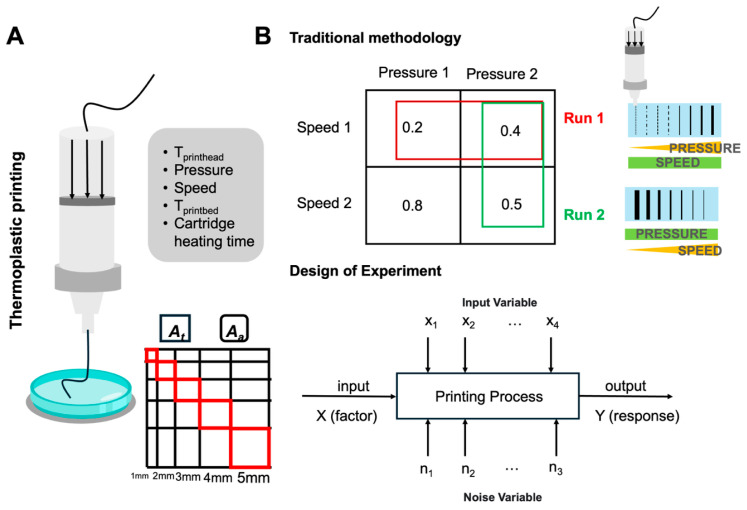
(**A**) Schematic representation of thermoplastic extrusion printing and the lattice-based filament fidelity model. A_t_ and A_a_ denote the theoretical and actual pore areas used to quantify dimensional accuracy. (**B**) Comparison between the traditional One-Factor-At-a-Time optimisation approach and the Design of Experiment methodology [31]. The lattice scores illustrate differences in filament quality under varying combinations of pressure and printing speed.

Extrusion-based 3D printing using granule feedstocks has emerged as a versatile additive manufacturing technique capable of producing complex geometries with high dimensional accuracy [1,2]. This layer-by-layer fabrication process involves the controlled deposition of thermoplastic materials through a heated nozzle, thereby enabling rapid prototyping and customised manufacturing across various industries [3,4]. In terms of biomedical applications, the extraordinary advantages that 3D printing may provide, such as its great control over the spatial organisation of biomaterials, repeatability, and overall simplicity, set it apart from other biofabrication approaches [5]. Despite its widespread adoption, the print quality of 3D-printed constructs is highly dependent on process parameters, especially when it comes to printing with new materials [6,7]. Optimising key parameters [8] such as temperature, speed, pressure, etc., and their combinations (Figure 1A) is a major problem that must be comprehensively explored, and which can take considerable time.

Polyhydroxybutyrate (PHB) is a biodegradable polyester derived from microbial fermentation and has demonstrated considerable potential in bone tissue engineering applications [9]. It exhibits excellent biocompatibility and provides mechanical support and structural stability owing to its high crystallinity [10]. In vivo, PHB can be metabolised into non-toxic by-products, ultimately degrading into carbon dioxide and water [11]. In addition, PHB can be blended with other biodegradable polymers, such as polycaprolactone (PCL), or reinforced with inorganic nanoparticles to improve its versatility and printability [12].

Representative studies have demonstrated the utility of PHB-related materials in tissue engineering. For example, Pecorini et al. [13] fabricated 3D-printed poly(3-hydroxybutyrate co 3-hydroxyvalerate) (PHBV)/poly(lactide co glycolide) composite scaffolds loaded with β tricalcium phosphate, which exhibited fully interconnected porous architectures and supported enhanced human mesenchymal stem or stromal cell viability, indicating strong potential for bone tissue regeneration. In addition, Krobot et al. [14] reported the successful fused deposition modelling of PHB and PCL blends, achieving favourable biocompatibility and mechanical properties suitable for bone tissue engineering and related medical applications.

Despite these advantages, PHB remains challenging for melt extrusion printing due to its intrinsic brittleness, poor melt processability, and narrow thermal processing window [15]. In contrast, polycaprolactone (PCL) is widely regarded as readily processable in pressure-driven extrusion systems owing to its low melting temperature and relatively broad printing window, with stable deposition commonly reported at melt temperatures of approximately 90–120 °C under pneumatic extrusion conditions, facilitating reliable printing over a wide range of processing parameters [16]. Poly(lactic acid) (PLA), while extensively studied and commonly used in extrusion-based additive manufacturing, is predominantly processed in filament-fed systems and typically exhibits a broad processing window [17], with nozzle temperatures in the range of 190–230 °C [18], resulting in generally good dimensional stability [19] despite its inherent brittleness and limited toughness. From a technical perspective, PHB offers higher stiffness and structural support compared with PCL, but is more brittle than PLA [14], indicating a trade-off between mechanical robustness and processability among these biopolymers. Compared with both PCL and PLA, PHB therefore requires more precise control of temperature and extrusion conditions to achieve acceptable dimensional fidelity. Consequently, whether using neat PHB or PHB-based blends, a systematic optimisation strategy is essential to enable reliable extrusion printing and reproducible scaffold fabrication.

Printability is a key indicator of extrusion-based 3D-printing performance, as it reflects how accurately a construct reproduces its digital design [20,21]. For thermoplastic extrusion, it is typically characterised by extrudability, filament continuity, and shape fidelity [21]. To quantify these features, several geometric assessment models are used [22]. Gradually expanding lattice patterns, which compare the printed area to the designed geometry, provide a systematic measure of dimensional accuracy across different feature sizes (Figure 1A). Bridge-span models, involving unsupported filament deposition over increasing distances, further evaluate melt strength and resistance to sagging. Together, these assessments offer a practical framework for benchmarking printing behaviour, particularly when optimising parameters for materials with narrow processing windows such as PHB.

Currently, the most commonly used experimental design methodology is One-Factor-At-a-Time (OFAT), which means only one factor is varied at a time [23,24]. As illustrated in Figure 1B, when speed 1 is set as constant and pressure as variable, two different levels of pressure are tested (run 1). Based on the score of the printed constructs, pressure 2 is the best pressure condition in this trial. Consequently, pressure 2 is set as constant, and then 2 different speeds are tested based on this (run 2). So, pressure 2/speed 2 is presumably our best parameter combination. Inevitably, speed 2/pressure 1 is missed due to the experimental design, and this combination is actually the best parameter combination. However, if speed 2 is the first parameter to be investigated, the result could be totally different. That is to say, the result is highly related to the first parameter that we choose.

DoE provides a structured statistical approach for optimising complex manufacturing processes by examining how multiple input variables influence a defined output response [25,26]. As illustrated in Figure 1B, the printing process can be viewed as a system in which controllable factors (x_1_, x_2_, …, x_4_) act as input variables (x_n_) and the measured printing quality serves as the response (Y), while unavoidable noise variables (n_1_, n_2_, n_3_), such as ambient fluctuations or material inconsistencies, may also affect the outcome [27]. Unlike OFAT, DoE enables several parameters to be varied concurrently, allowing both dominant factors and interaction effects to be identified [28]. This results in empirical models capable of predicting print performance and locating optimal parameter combinations with substantially reduced experimental effort [29].

In melt extrusion 3D printing, DoE is particularly valuable because printing quality depends on coupled thermal and mechanical conditions [30]. Accordingly, five controllable factors, printhead temperature (T_printhead_), extrusion pressure, printing speed, bed temperature (T_printbed_), and cartridge heating time, were selected as the primary determinants of PHB printability. Given the dimensionality of this parameter space, a fractional factorial design was adopted to explore the system efficiently whilst maintaining statistical robustness.

Hence, in this study, bridge and lattice test models were employed to comprehensively assess the printability of PHB scaffolds. By developing a quantitative rating framework, we were able to objectively compare constructs produced under different parameter combinations and identify the conditions that most strongly influenced printing performance. This approach provides a clear and reproducible route for determining optimal extrusion parameters, thereby supporting more reliable fabrication of PHB-based scaffolds.

## 2. Materials and Methods

### 2.1. Materials

Polyhydroxybutyrate (PHB, average molecular weight 550 kg/mol, 5 mm granules, Goodfellow Cambridge Limited, Huntingdon, UK) was used in this study. The material was dried at 70 °C for at least 1 h before printing. According to the manufacturing note, this PHB contains up to 2 wt% of 3-hydroxyvalerate (3HV) units, which arise naturally during microbial synthesis. Therefore, in the following sections, this material is referred to as poly(3-hydroxybutyrate-co-3-hydroxyvalerate) (PHBV, ~2 wt% HV) [32].

### 2.2. Differential Scanning Calorimetry (DSC)

DSC was performed using a DSC Q100 instrument (TA Instruments, Newcastle, DE, USA) to investigate the thermal properties of PHBV granules by which assess the range of the printing temperature. DSC scans were carried out between 60 and 240 °C with a 10 °C·min^−1^ heating rate. Prior to the measurements, the DSC cell was calibrated using indium with 20 °C·min^−1^ heating rate under a 50 cm^3^·min^−1^ nitrogen atmosphere. Then, 5–10 mg of samples (*n* = 3) was applied in each measurement. The enthalpy of fusion (ΔHf) was obtained from the corresponding DSC run and used to quantify the degree of crystallinity (Xc%), according to the following equation:

(1) $$x_{c}(\%)=\frac{{\Delta H}_{f}}{{\Delta H}_{f}^{0}}*100\%$$

where ${\Delta H}_{f}^{0}$ is the melt enthalpy of a 100% crystalline PHBV sample (146 J/g [33]).

### 2.3. Extrusion Printing Setup and Parameters

Extrusion printing was performed using a BIO X6 printer (CELLINK, Gothenburg, Sweden) equipped with a thermoplastic printhead operating in a pneumatic extrusion mode. The thermoplastic printhead provides a heating capacity of up to 250 °C, enabling the processing of thermoplastic materials. In this study, PHBV granules were printed using an original manufacturer-supplied stainless-steel thermoplastic cartridge (10 mL) fitted with an exchangeable thermoplastic nozzle with an inner diameter of 0.4 mm. For each printing run, 1 g of PHBV granules was loaded into the cartridge. Printing was performed on a glass Petri dish serving as the build substrate.

In this study, the selected printing parameters represent key process variables governing material flow, deposition, and shape retention. Specifically, the printhead temperature, defined as the nozzle heating temperature, and the extrusion pressure, defined as the applied pneumatic pressure driving material flow, jointly control melt viscosity and flow behaviour; the printing speed, corresponding to the nozzle translation speed during deposition, influences filament stretching and deposition accuracy; and the printbed temperature, defined as the substrate temperature, affects interfacial adhesion and cooling behaviour.

Prior to printing, the cartridge was heated using the integrated printhead heater. The cartridge heating time refers to the preheating duration applied to the material-filled cartridge before extrusion, allowing the PHBV granules to melt and reach thermal equilibrium at the set printhead temperature. During printing, extrusion was driven by an external pneumatic pressure regulator, and the extrusion process was visually monitored to ensure continuous material flow.

### 2.4. DoE Setup

The DoE model used was fractional factorials, with two levels (low (−1) and high (+1)) and three centre points within one block. For a five-factor design, 16 runs with one centre point can achieve a resolution of V, meaning that the main factors and two combinations are not confounded. Based on pre-experiments, values used for low- and high-level settings for PHBV printing were as follows: T_printhead_: 145 °C (−1)/165 °C (+1), pressure at 150: (−1)/170 kPa (+1), speed: 3 (−1)/15 mm/s (+1), T_printbed_: 25 (−1)/60 °C (+1), cartridge heating time: 0 (−1)/120 s (+1). To increase the accuracy of the model, a 16-run experiment with 3 centre points was generated. The effects and interactions of these parameters on printing performance and dimensional fidelity were subsequently analysed.

### 2.5. Printability Assessment

While the bridge model (see Supplementary Material Figure S1), which describes the mechanical stability of a suspended filament by balancing gravitational forces and the material’s viscoelastic response, was used to assess extrudability, the filament fusion test (FFT) was implemented to evaluate filament formation and printability [34]. In this context, FFT quantifies post-deposition material spreading by analysing the fusion behaviour of neighbouring filaments or deviations between theoretical and experimentally measured pore geometries in PHBV scaffolds. The parameter of the lattice is shown in Figure 1A. Image J 1.53a (National Institutes of Health, Bethesda, MD, USA) was used to measure the actual length/area of printed scaffolds. Scaffold images were calibrated using a ruler as a known reference length, after which the enclosed pore area defined by adjacent printed filaments in the filament fusion test (see Figure 1A, highlighted square area in red) was measured. All measurements were performed in triplicate and compared with the theoretical pore area.

In this study, printability was evaluated using geometrical descriptors that characterise post-deposition filament spreading and shape fidelity of the printed lattice. The printability ($P_{r}$) represents the ability of the printed structure to reproduce the designed enclosed pore geometry. The previously derived equation for $P_{r}$ is given as follows [35,36].

(2) $$P_{r}=\frac{L^{2}}{{16A}_{c}}$$

where *L* is the enclosed pore area perimeter, *A_c_* is the enclosed pore area. A printed structure was considered to exhibit good printability when the calculated value $P_{r}$ was close to that of the theoretical lattice, indicating high fidelity in reproducing the designed geometry.

### 2.6. Image Analysis and Geometrical Measurements

Geometrical measurements of the printed lattice structures were performed using Image J 1.53a (National Institutes of Health, Bethesda, MD, USA) [37]. All images were first opened in ImageJ and calibrated based on the embedded scale bar. Specifically, a straight-line selection was drawn along the scale bar, and the image scale was set via Analyse → Set Scale by inputting the known distance and the corresponding unit of length.

After scale calibration, straight-line selections were used to measure characteristic lengths of the printed lattice, including the perimeter segments of the enclosed pore area. Length measurements were obtained using Analyse → Measure. The measured lengths were subsequently used to determine the perimeter *L* required for the calculation of the printability parameter *P*_r_.

Area measurements were conducted on the same scale-calibrated images. For enclosed pore areas with regular geometries, the rectangle selection tool was used to define the region of interest, followed by Analyse → Measure to obtain the enclosed area. For irregular enclosed pore areas, the polygon selection tool was employed to manually trace the pore boundary, and the enclosed area was subsequently determined using Analyse → Measure. This approach allows accurate determination of enclosed pore areas for both regular and non-rectangular geometries without assuming an idealised pore shape.

All measurements were performed by the same operator following an identical and consistent procedure to minimise operator-dependent variability.

### 2.7. Statistical Analysis

Minitab^®^ 19.1 (64-bit) (Minitab, LLC , State College, PA, USA) was used for statistical analysis. After data had been gathered, a stepwise backwards elimination was used to analyse the outcome. Although the model was compelled to retain single factors, this process was repeated until it contained only factors with a *p*-value < 0.15. In exploratory DoE analyses, relaxed significance levels (e.g., *p* < 0.10–0.15) are commonly used during factor screening to avoid prematurely excluding relevant variables when the number of experimental runs is limited [27].

## 3. Results

### 3.1. Thermal Properties of PHBV Granules

**Figure 2:**
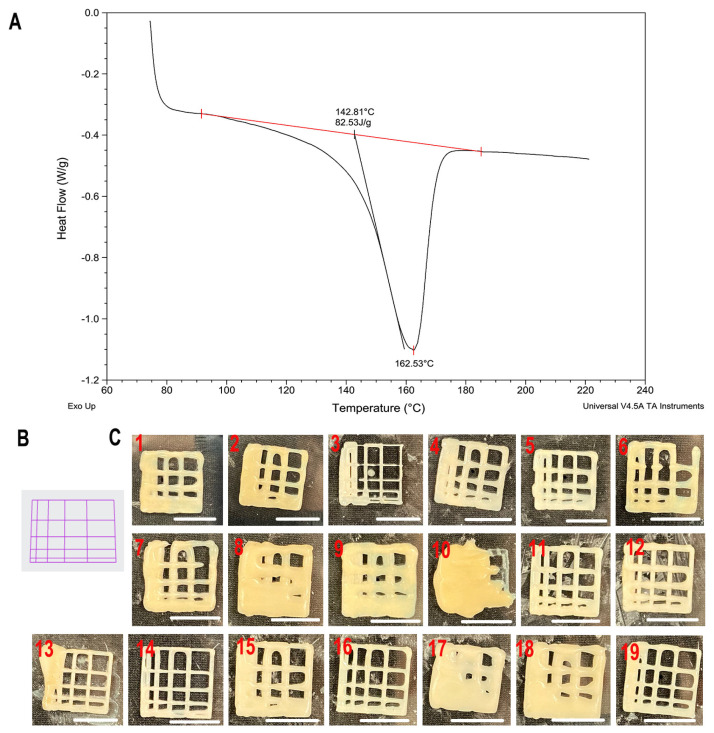
(**A**) DSC thermogram of PHBV granules showing the melting endotherm and corresponding melting temperature. The red baseline and markers indicate the determination of the melting enthalpy (ΔH = 82.53 J/g). The onset melting temperature (T_onset_ = 142.81 °C), peak melting temperature (T_m_ = 162.53 °C), and endset melting temperature (T_endset_ ≈ 178 °C) are indicated. Exothermic direction is upward. (**B**) G-code-derived printing toolpath representation of the grid structure. (**C**) Representative images of the printed lattice constructs obtained from all parameter combinations (RunOrder 1–19), illustrating variations in filament formation and pore fidelity. Scale bar: 1 mm.

The DSC result showed that the melting point (Tm) of PHBV granules was 143 °C (Figure 2A). The phase transition was completed at 163 °C, after which the material returned to baseline heat flow behaviour. This temperature range is critical for defining the processing window for extrusion-based printing.

To ensure sufficient melting and enable material flow through the nozzle, the printing temperature must exceed the melting onset temperature. In preliminary printing trials, temperatures below 145 °C resulted in incomplete melting of the PHBV granules and unstable or interrupted extrusion. Conversely, when the temperature exceeded 165 °C, the melt viscosity decreased excessively, leading to unstable, surge-like extrusion and severe post-deposition spreading, which prevented the printed lattice from retaining its designed geometry. Therefore, the printing temperature range for the experimental design was selected as 145–165 °C, representing a stable and reproducible printing window that balances material flow and shape fidelity.

The temperature of the printbed is also another factor to be considered. This is highly related to the adhesion behaviour of the printed constructs to the printing media, such as a glass Petri dish. Warping happens during cooling as PHBV molecules reorganise into tightly packed crystalline regions. This shrinkage generates internal stresses within the printed part during printing multi-layer structures. Based on Juan et al.’s research, it can only achieve a good adhesion when the temperature goes above 60 °C [38]. On the other hand, the degree of crystallinity can be calculated as approximately 57% based on the enthalpy of fusion (83 J/g). Such a relatively high crystallinity was associated with a narrow range of stable printing temperatures. Crystallisation during cooling was accompanied by dimensional shrinkage, which manifested as pore size reduction and increased sensitivity to printing and bed temperature. These observations indicate that the crystallinity of PHBV is an important factor influencing the selection and optimisation of printing parameters.

### 3.2. Design of Experiment

A total of 19 experimental runs were generated according to the Design of Experiment, as summarised in Table 1. Each parameter combination was used to print the proposed assessment models.

The results of the bridge model test for all runs are presented in Figure S1. No discernible differences in filament sagging or collapse behaviour were observed between individual runs based on qualitative visual inspection. As a result, the bridge model did not provide sufficient sensitivity to distinguish between the investigated processing conditions and was therefore excluded from further quantitative analysis.

All 19 runs of the printed lattice model are shown in Figure 2C, and the corresponding quantified results are summarised in Table 2. For clarity, the analysis focused exclusively on the enclosed square areas formed along the diagonal direction (Figure 1A, red outlined area). The nominal 1 × 1 mm square was excluded from the analysis, as its formation was primarily limited by the printer resolution, nozzle diameter, and intrinsic material behaviour rather than by the investigated processing parameters.

Compared with the G-code-derived printing toolpath (Figure 2B), the printed grids obtained under the 19 parameter combinations (Figure 2C) exhibited recurring fabrication defects typical of extrusion-based printing. Across the parameter space, thread heterogeneity was frequently observed, manifested as variations in filament width, local spreading or flattening, and occasional thinning or partial discontinuities. The extent of these non-uniformities varied among parameter combinations, indicating that filament formation was sensitive to the selected printing conditions, particularly at turning points and filament intersections.

Printing-induced shrinkage was also evident in multiple parameter sets, resulting in a reduction in enclosed pore area and, in some cases, in the overall grid dimensions compared with the designed toolpath. As a combined consequence of filament heterogeneity and shrinkage, deviations from the designed enclosed pore geometry (pore geometry violations) were observed, including distorted pore outlines, rounded corners, irregular pore sizes, and occasional partial pore blockage or pore merging. In the most severe cases, local collapse and loss of open pores were observed, whereas several parameter combinations still preserved an overall lattice-like architecture with open and distinguishable pores.

The fractional factorial design was applied to screen out the non-distinctive interactive combinations, leaving a significant model. The elimination process started by assessing the two-factor combination with the highest *p*-value; the single main factor was kept within the model until all the *p*-values of the parameter combinations were less than 0.15 (Table 3), in line with previous work [39].

**Figure 3:**
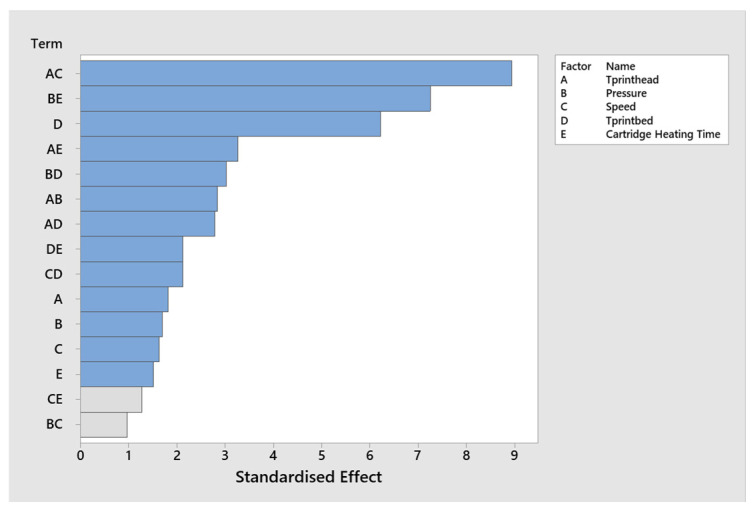
Pareto chart showing the standardised effects of the main factors and their interactions on printability. Grey bars indicate terms that were not retained in the final regression model. A: T_printhead_, B: Pressure, C: Speed, D: T_printbed_, E: Cartridge heating time.

The Pareto chart illustrated the extent to which individual or combination factors influence the measured output, represented in standardised effect. It refers to the magnitude of a factor’s influence on the measured response after normalising by its standard error. As shown in Figure 3, AC (T_printhead_ × Speed) and BE (Pressure × Cartridge Heating Time) had the largest standardised effect, suggesting a strong interaction between those factor combinations on the printability. The effect of D (T_printbed_) is also substantial and much larger than the one measured with the other factors (A–C, E), showing that this factor independently affects the response.

**Figure 4:**
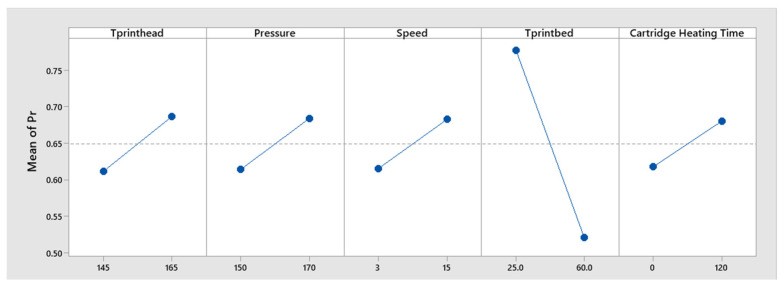
Main effects plot for printability, showing the influence of each parameter included in the final regression model.

The main effects plots illustrated the direction of an effect and were valuable for evaluating whether major factors had a positive or negative influence on measured output. According to Figure 4, the temperature of the printhead, pressure, speed and cartridge heating time had a positive effect on the printability model, whereas the temperature of printbed had a negative effect in the final DoE model.

**Figure 5:**
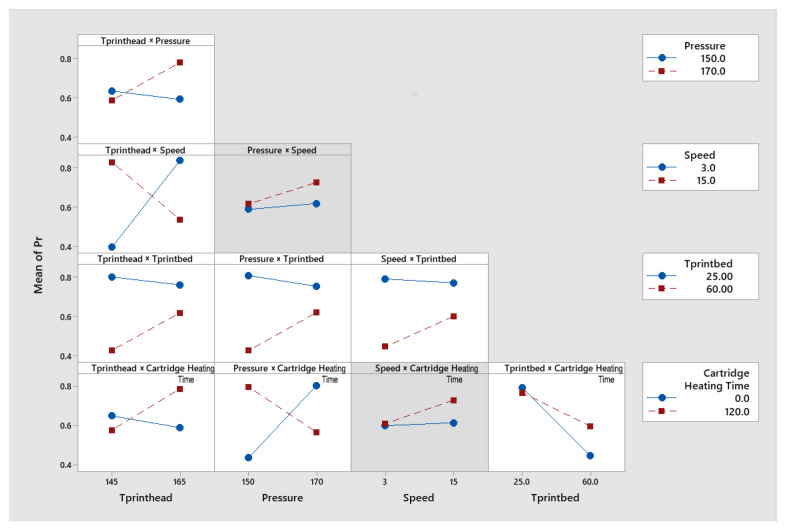
Interaction plot for printability, illustrating the combined effects of paired factors. Panels with a grey background indicate interaction terms that were not retained in the final model.

The interaction plot illustrated whether specific combinations of factors had synergistic effects, either positively or negatively. Typically, two lines were represented on a graph illustrating the low and high levels of a variable. Figure 5 displays the interaction between two main factors, and the significant combination had been highlighted to show their tendency (in the model) to influence the printing construct. For example, as shown in the pressure–cartridge heating time plot, when the cartridge heating time was held at 120 s, the mean value of printability decreased with increasing pressure. Conversely, when the heating time was set at 0 s, the mean value of printability increased. This reminded us of the optimisation of the parameters. The possible explanation could be that the flow was low when the temperature of the nozzle just reached the set temperature, due to either the fact that the PHBV granule was not completely melted or that the viscosity of the ink was too high, so that higher pressure was needed to enable continuous extrusion.

**Figure 6:**
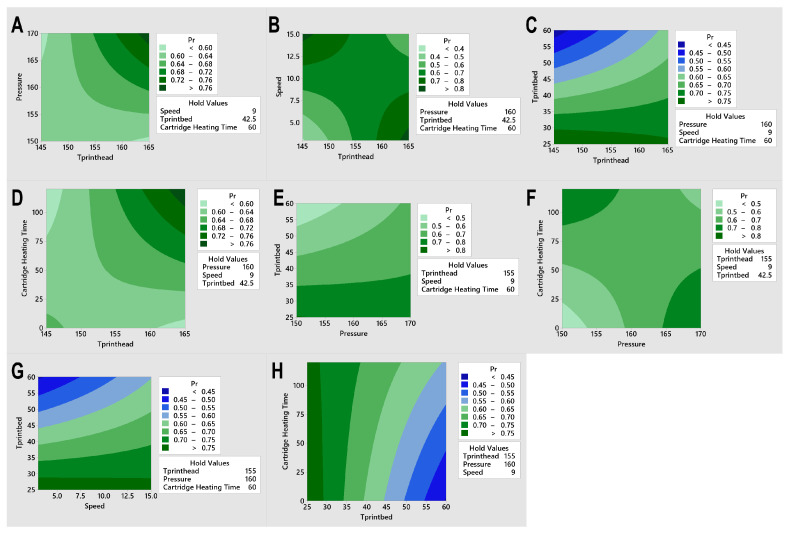
Contour plots illustrating the two-factor interaction effects on the printability response across varying combinations of processing parameters. (**A**) Effect of T_printhead_ and pressure at fixed speed (9 mm/s), T_printbed_ (42.5 °C), and cartridge heating time (60 s). (**B**) Effect of T_printhead_ and speed at fixed pressure (160 kPa), T_printbed_ (42.5 °C), and cartridge heating time (60 s). (**C**) Effect of T_printhead_ and T_printbed_ at fixed pressure (160 kPa), speed (9 mm/s), and Cartridge heating time (60 s). (**D**) Effect of T_printhead_ and cartridge heating time at fixed pressure (160 kPa), speed (9 mm/s), and T_printbed_ (42.5 °C). (**E**) Effect of pressure and T_printbed_ at fixed T_printhead_ (155 °C), speed (9 mm/s), and cartridge heating time (60 s). (**F**) Effect of pressure and cartridge heating time at fixed T_printhead_ (155 °C), speed (9 mm/s), and T_printbed_ (42.5 °C). (**G**) Effect of speed and T_printbed_ at fixed T_printhead_ (155 °C), pressure (160 kPa), and cartridge heating time (60 s). (**H**) Effect of T_printbed_ and cartridge heating time at fixed T_printhead_ (155 °C), pressure (160 kPa), and speed (9 mm/s).

Contour plots were given to graphically depict the design space of two interacting factors as well as how to fully optimise the factor settings for the intended outcome. Figure 6 shows the two-parameter interactions and how they influenced the values of printability, which were categorised in the plot in coloured regions (from green to blue). The lowest printability was with the lightest green or darkest blue. Constant parameter values were listed as a legend, i.e., when we were analysing the interaction between pressure and cartridge heating time, we were holding other parameters as constant. In this way, we could clearly know that the optimal factor combination appeared at the two corners.

Based on the elimination process, a regression equation was generated as shown below.

(3) $$\begin{array}{cc} Pr=13.51 & -0.0832T_{printhead}-0.0877Pressure+0.4658Speed-0.1217T_{printbed}\\ & +0.02131Cartridage\;Heating\;Time+0.000588T_{printhead}\times Pressure\\ & -0.003083T_{printhead}\times Speed+0.000329T_{printhead}\times T_{printbed}\\ & +0.000113T_{printhead}\times Cartridge\;Heating\;Time+0.000357Pressure\times T_{printbed}\\ & -0.000250Pressure\times Cartridge\;Heating\;Time+0.000417Speed\times T_{printbed}\\ & +0.000042T_{printbed}\times Cartridge\;Heating\;Time \end{array}$$

The multiple response prediction using maximum goal was T_printhead_ at 145 °C, Pressure at 150 kPa, Speed at 15 mm/s, T_printbed_ at 25 °C, and Cartridge Heating Time at 120 s.

**Figure 7:**
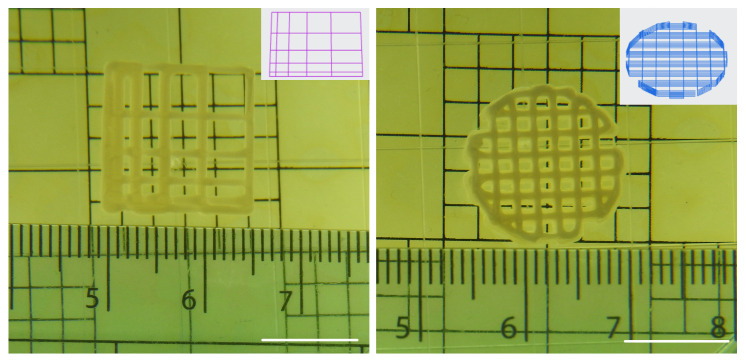
Printed lattice construct (**left**) and the corresponding G-code-derived scaffold (**right**) produced using the optimised parameter set generated from the DoE analysis. Scar bar: 1 cm.

Figure 7 illustrates the successfully printed lattice (left) and multi-layer scaffold (right) fabricated using the optimised parameter combination. The lattice demonstrates a uniform grid structure with well-defined filaments and consistent enclosed pore area, indicating high dimensional accuracy and good layer adhesion. The calculated printability of 0.97 ± 0.02 confirms the fidelity between the designed and printed geometries.

To further validate the feasibility of multi-layer fabrication, the same parameters were used to print a stacked scaffold (right). The printed construct retained its designed pattern without visible deformation or filament collapse, suggesting sufficient interlayer fusion and mechanical stability. The use of double-sided tape effectively prevented warping during printing at the optimal printbed temperature of 25 °C, ensuring good adhesion of the initial layer. The inset schematic shows the designed grid pattern used for scaffold prints.

## 4. Discussion

The present study establishes a systematic and reproducible framework for optimising the extrusion-based printing of PHBV, a biopolymer that is well-recognised for its narrow processing window and sensitivity to thermal and rheological conditions [15,40]. By combining DSC with a DoE-based parameter screening approach, a stable printing window was identified in which material flow, dimensional fidelity, and pore integrity could be balanced. This integrated strategy directly links the thermal behaviour of PHBV with extrusion performance, providing a practical basis for process selection when fabricating lattice-type scaffolds.

From a tissue engineering perspective, precise control over filament deposition and pore geometry is essential, as scaffold architecture strongly influences mechanical stability, porosity, and the ability to support cell infiltration and tissue ingrowth [41,42]. The optimised parameter set identified in this work therefore offers a practical guideline for the fabrication of PHBV scaffolds with reproducible geometries, reducing empirical trial-and-error during process development. More broadly, the DoE-driven optimisation strategy demonstrated here is transferable to PHBV-based composites or related biodegradable thermoplastics, facilitating the rational design of extrusion-printed scaffolds for biomedical applications [43,44].

The thermal behaviour and printability observed in this study can be contextualised by considering the role of hydroxyvalerate (HV) units in PHBV. It is well-established that the incorporation of HV disrupts the regular packing of PHB chains [45], acting as an internal plasticising comonomer that lowers the melting temperature and reduces the degree of crystallinity relative to neat PHB [46,47]. These changes are accompanied by improved ductility and reduced brittleness, which are advantageous for extrusion-based processing [48,49]. In addition, the reduction in crystallinity associated with HV incorporation has been reported to mitigate shrinkage and warping by alleviating residual stresses generated during cooling [17,50]. These established effects help explain the existence of a comparatively narrow but usable printing window identified in the present work.

In comparison with previous studies on PHB- and PHBV-based extrusion printing, the optimised processing window obtained here falls within the lower end of the temperature ranges typically reported for melt extrusion of polyhydroxyalkanoates, where excessive temperatures often lead to melt instability, post-deposition spreading, and loss of geometric fidelity [13]. Similar challenges related to temperature sensitivity and dimensional instability have been widely reported, frequently necessitating either material modification or extensive parameter tuning [51]. In agreement with this literature, the present DoE analysis identified printhead temperature and pneumatic pressure as dominant factors governing filament formation and pore geometry, reinforcing the necessity of systematic optimisation when processing PHB-derived melts.

With respect to printability assessment, bridge-type models have been widely used as qualitative tools to evaluate extrudability and filament stability, particularly for hydrogel-based or viscoelastic inks [20,52]. However, under the conditions investigated here, the bridge model did not provide sufficient sensitivity to discriminate between processing parameters for molten PHBV. This limitation is likely attributable to the relatively high melt viscosity and limited rheological variation within the selected parameter space. In contrast, filament fusion-based metrics, which directly quantify post-deposition spreading and pore- or enclosed-area deviation, proved more effective for assessing geometric fidelity and have been widely adopted in extrusion-printing literature [53,54]. These observations highlight the importance of selecting printability metrics that are appropriate for the rheological regime of the material under investigation.

Several limitations of the present methodology should nevertheless be acknowledged. The statistical analysis employed a relaxed significance threshold (*p* < 0.15) as part of an exploratory DoE screening strategy, reflecting a deliberate balance between statistical stringency and the risk of overlooking influential parameters in a limited experimental design [55]. In addition, the study focused on single-layer lattice structures to enable systematic evaluation of parameter effects. When extending the approach to multi-layer scaffolds, additional factors such as interlayer bonding [56], layer stacking stability, and cumulative thermal history [57] are expected to play a significant role and will require further optimisation. Moreover, although stable extrusion and reproducible filament geometry were observed when processing PHBV granules, no dedicated quantitative analysis of internal voids or melt homogeneity was performed [58,59]. Future studies incorporating imaging-based characterisation or degassing-assisted processing could provide deeper insight into these aspects.

Overall, this work demonstrates that coupling thermal analysis with a DoE-based optimisation strategy provides an effective route to address the intrinsic processing challenges of PHBV. The findings contribute practical guidance for extrusion-based fabrication of PHBV scaffolds and lay the groundwork for future studies on multi-layer architectures, PHBV-based blends or composites, and biologically functional scaffold systems.

## 5. Conclusions

This study applied a fractional factorial Design of Experiment (DoE) approach to identify the optimal extrusion printing parameters for PHBV, a thermoplastic polymer widely used in scaffold fabrication. Five key factors were examined, and fewer than twenty experimental runs were required to construct a significant empirical model describing their influence on printability. DSC analysis validated the thermal properties of the PHBV granules and supported the selection of appropriate factor levels. Within the tested ranges, printhead temperature, pressure, printing speed and cartridge heating time improved lattice fidelity, whereas bed temperature had an adverse effect, and several synergistic interactions, such as between cartridge heating time and pressure, were identified. Using the DoE-derived optimal parameter combination (T_printhead_ 145 °C, pressure 150 kPa, speed 15 mm/s, T_printbed_ 25 °C and cartridge heating time 120 s), both lattice and scaffold models were successfully fabricated, demonstrating the practical value of the optimised conditions. Overall, this work provides a systematic and quantitatively validated pathway for PHBV printability optimisation and establishes a methodological framework that can be readily extended to other thermoplastic biomaterials.

## Figures and Tables

**Table 1:** Full parameter combinations used for evaluation test generated by minitab.

StdOrder	RunOrder	CenterPt	Blocks	T_printhead_/°C	Pressure/kPa	Speed/mm/s	T_printbed_/°C	Cartridge Heating Time/s
8	1	1	1	165	170	15	25	0
4	2	1	1	165	170	3	25	120
5	3	1	1	145	150	15	25	0
1	4	1	1	145	150	3	25	120
16	5	1	1	165	170	15	60	120
14	6	1	1	165	150	15	60	0
9	7	1	1	145	150	3	60	0
2	8	1	1	165	150	3	25	0
18	9	0	1	155	160	9	42.5	60
11	10	1	1	145	170	3	60	120
12	11	1	1	165	170	3	60	0
19	12	0	1	155	160	9	42.5	60
17	13	0	1	155	160	9	42.5	60
15	14	1	1	145	170	15	60	0
7	15	1	1	145	170	15	25	120
10	16	1	1	165	150	3	60	120
6	17	1	1	165	150	15	25	120
3	18	1	1	145	170	3	25	0
13	19	1	1	145	150	15	60	120

**Table 2:** Actual area and perimeter measured by Image J and calculated printability.

Run Order	Actual Area/mm^2^	Perimeter/mm	Printability
1	8.65	9.66	0.67
2	8.97	10.93	0.83
3	15.34	15.10	0.93
4	8.92	10.39	0.76
5	9.05	10.02	0.69
6	0.00	0.00	0.00
7	0.00	0.00	0.00
8	6.59	8.92	0.75
9	4.80	7.34	0.70
10	0.00	0.00	0.00
11	11.56	12.79	0.88
12	10.63	11.13	0.73
13	14.97	13.16	0.72
14	11.46	12.47	0.85
15	10.20	10.58	0.69
16	10.79	12.07	0.84
17	5.11	7.72	0.73
18	6.08	6.08	0.76
19	11.46	12.08	0.80

**Table 3:** Backward stepwise elimination of five printing parameters and their two-factor combinations. The coloured row represents the elimination sequence; the *p*-value of 0.15 is applied.

	Step 1		Step 2		Step 3	
Term	Coef	*p*-Value	Coef	*p*-Value	Coef	*p*-Value
Constant	0.6489		0.6489		0.6489	
T_printhead_	0.0375	0.139	0.0375	0.120	0.0375	0.130
Pressure	0.0350	0.158	0.0350	0.140	0.0350	0.151
Speed	0.0338	0.169	0.0338	0.151	0.0338	0.164
Tprintbed	−0.1288	0.006	−0.1288	0.002	−0.1288	0.002
Cartridge Heating Time	0.0312	0.193	0.0312	0.176	0.0312	0.191
T_printhead_ ×Pressure	0.0587	0.052	0.0587	0.037	0.0587	0.036
T_printhead_ × Speed	−0.1850	0.002	−0.1850	0.001	−0.1850	0.000
T_printhead_ × T_printbed_	0.0575	0.054	0.0575	0.039	0.0575	0.039
T_printhead_ × Cartridge Heating Time	0.0675	0.037	0.0675	0.024	0.0675	0.022
Pressure × Speed	0.0200	0.363				
Pressure × T_printbed_	0.0625	0.044	0.0625	0.030	0.0625	0.029
Pressure × Cartridge Heating Time	−0.1500	0.004	−0.1500	0.001	−0.1500	0.001
Speed × T_printbed_	0.0438	0.101	0.0438	0.083	0.0438	0.088
Speed × Cartridge Heating Time	0.0262	0.255	0.0262	0.240		
T_printbed_ × Cartridge Heating Time	0.0438	0.101	0.0438	0.083	0.0438	0.088
		R-sq				
		97.84%		Eliminations:	1st	2nd

## Data Availability

The original contributions presented in the study are included in the article; further inquiries can be directed to the corresponding authors.

## Supplementary Materials

The following supporting information can be downloaded at https://www.mdpi.com/article/10.3390/jfb17020090/s1, Figure S1: Images of all printed constructs based on the bridge model.
