# Supplementary material for: Parameter Optimisation in 3D Extrusion Printing of Polyhydroxybutyrate Using Design of Experiment Methodology
Source: J Funct Biomater. 2026 Feb 12;17(2):90. doi: 10.3390/jfb17020090 (PMC12941641; doi:10.3390/jfb17020090)
Supplement: Supplementary file 1 [file jfb-17-00090-s001.zip › jfb-4111494-supplementary.pdf]

Appendices: Supplementary Information

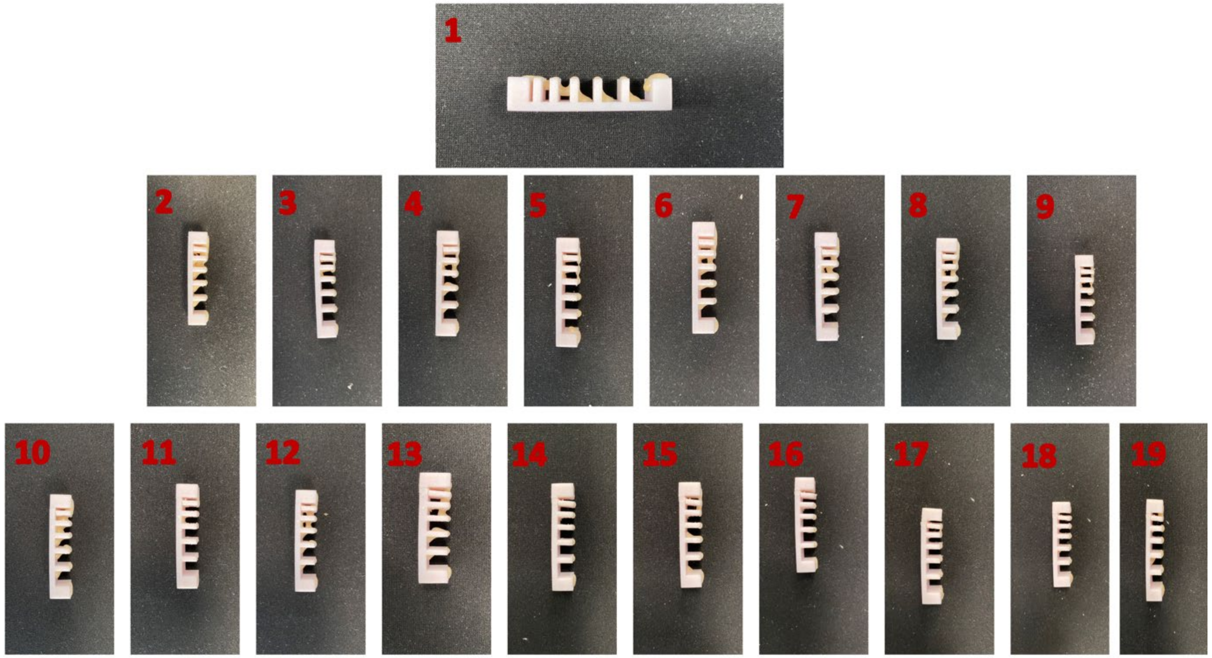

Figure S1. Images of all printed constructs based on the bridge model.
